# Blister Pack Ingestion in an Elderly Patient With a Communication Barrier: A Case Report

**DOI:** 10.7759/cureus.40968

**Published:** 2023-06-26

**Authors:** Yuan-Zhao Zhu, Yan-Lin Pu, Hui-Qiao Chen, Li-Hua Li

**Affiliations:** 1 Department of Gerontology, The First Affiliated Hospital of Dali University, Dali, CHN; 2 Department of Internal Medicine, Fugong People's Hospital, Fugong, CHN

**Keywords:** nursing, management, communication barrier, elderly, foreign body ingestion

## Abstract

Foreign body ingestion is a common problem among elderly patients and can pose a serious health risk, particularly for those with communication barriers, cognitive impairments, or obscure medical histories. This report presents the case of a 67-year-old female inpatient who had a language communication barrier and accidentally ingested a blister pack. Effective communication was facilitated through an interpreter, and prompt endoscopic intervention was conducted to remove the foreign body safely. The patient was discharged with no further symptoms during follow-up. This case highlights the importance of prompt evaluation and intervention for foreign body ingestion in elderly patients, particularly those with communication barriers.

## Introduction

Compared to children, the incidence of foreign body ingestion in adults is low [1]. In 95% of cases, foreign body ingestion is accidental and often associated with food, such as fish and chicken bones or toothpicks [2]. Foreign body ingestion is commonly observed in older adults, individuals with underlying psychiatric diseases or alcohol intoxication, prison inmates, and those who ingest foreign bodies for drug trafficking [3-5]. Foreign body ingestion poses a significant health concern in the elderly population due to potentially severe complications such as airway obstruction, gastrointestinal bleeding, systemic infection, gastrointestinal tract perforation, or aspiration-induced lung damage [6-8]. In this report, we present a case of foreign body ingestion in an elderly patient with a language communication barrier, discuss the common reasons for foreign body ingestion, and recommend appropriate management strategies.

## Case presentation

**Figure 1:**
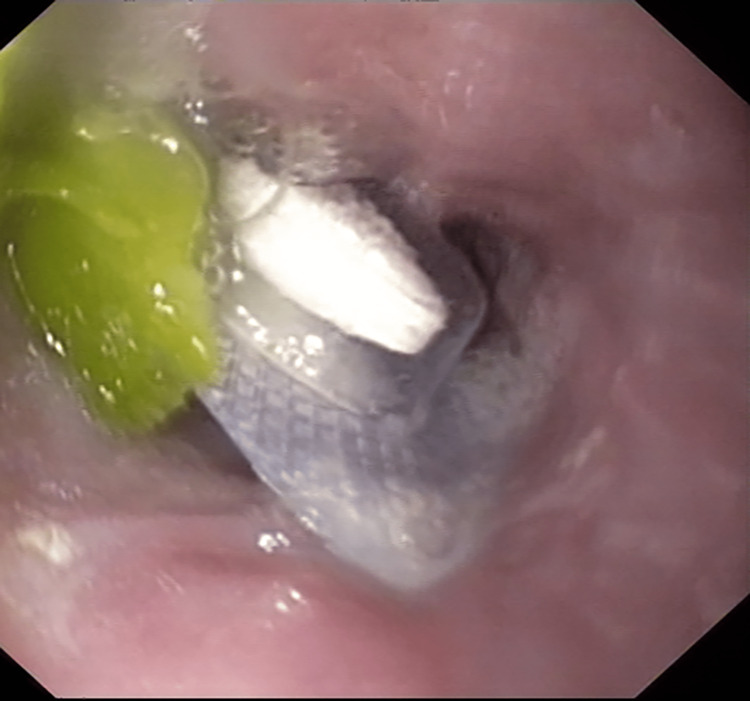
An emergency gastroscopy revealed a blister pack located in the esophagus.

We present a case of foreign body ingestion in a 67-year-old female patient from the Lisu ethnic group in Fugong County, Nujiang Prefecture, Yunnan Province. The patient was widowed, and neither she nor her accompanying family members spoke Chinese, which created communication barriers during medical care. The patient had been hospitalized for cough, phlegm, and shortness of breath for one week and had a long-standing history of hypertension without antihypertensive treatment, newly diagnosed hyperlipidemia, and type 2 diabetes. On the fifth day of hospitalization, the nurse dispensed the patient's nighttime medications, including blister-packing atorvastatin calcium. The nurse did not follow the medication administration principle of "properly instructing the patient and ensuring the medication is swallowed" before leaving the patient's room. The patient accidentally ingested all her medications, including the blister-packing atorvastatin calcium, as she could not understand Mandarin and felt drowsy while her family members were asleep. Later that night, the patient experienced discomfort in the throat, but due to communication difficulties, she only received palliative treatment for her symptoms. The next morning, with the translator's help, it was understood that the patient had accidentally ingested a blister pack, and a CT scan of the neck was performed. The CT scan of the neck showed a high-density shadow in the anterior strip of the sixth cervical vertebra, suggesting the possible presence of a blister pack. Further emergency gastroscopy confirmed the blister pack (Figure 1), and the gastroenterologist carefully removed the blister pack. The patient had no complications such as bleeding or perforation, and the discomfort symptoms quickly improved.

## Discussion

Foreign body ingestion can be more challenging when the patient has communication barriers, cognitive impairment, or an insufficient understanding of medication guidance. In this case, the patient had limited proficiency in Mandarin, which created communication barriers during medical care. The patient accidentally ingested blister-packing atorvastatin calcium, which caused discomfort in the throat and required further examination by an emergency gastroscopy to remove the blister pack.

There are several common reasons for foreign body ingestion in elderly patients, including poor vision, cognitive impairment, poor dentition, decreased mobility, and medication-related factors [9-11]. In this case, the accidental ingestion of the blister pack could have been caused by a combination of factors, including the patient's limited proficiency in Mandarin and her drowsy state at the time. It should also be noted that the patient was illiterate and unable to read the instructions on the packaging. Furthermore, the nurse failed to adhere to the hospital's management standards of properly instructing the patient and ensuring the medication was swallowed, contributing to this unfortunate event.

Appropriate management strategies for foreign body ingestion depend on the type and location of the foreign body and the patient's overall health status [8,9,12,13]. Most ingested foreign bodies (80%-90%) typically pass through the digestive system without any intervention [14]. However, around 10% to 20% of patients may require endoscopic intervention, while surgical intervention is only needed in less than 1% of cases [13,15]. In this case, an emergency gastroscopy was performed to remove the blister pack, and the patient's symptoms improved after treatment. It is important to note that foreign body ingestion can lead to potentially severe complications such as airway obstruction, gastrointestinal bleeding, systemic infection, perforation of the gastrointestinal tract, or aspiration-induced lung damage [7,8,12]. Therefore, timely diagnosis and treatment are crucial to preventing adverse outcomes [13,16].

This case highlighted several areas for improvement in medication management and administration. One of the key solutions to preventing foreign body ingestion is prevention through proper patient education and instruction, medication reconciliation, and monitoring of medication administration. Hospitals should focus on enhancing staff education and training on medication management and administration, improving medication reconciliation processes, and increasing patient education and engagement to prevent similar errors. Additionally, hospitals should recognize the need to provide language services in minority areas where language barriers may exist. This includes providing translators or volunteers who speak minority languages to help overcome language barriers and improve communication between healthcare providers and patients. By implementing these measures, we can improve patient safety and prevent medication errors, particularly in areas where language barriers may exist.

## Conclusions

Foreign body ingestion is a significant health concern for elderly patients, especially those with communication barriers or cognitive impairment. Healthcare providers should know the common reasons for foreign body ingestion and implement appropriate management strategies to prevent adverse outcomes.
